# Triciribine attenuates pathological neovascularization and vascular permeability in a mouse model of proliferative retinopathy

**DOI:** 10.1016/j.biopha.2023.114714

**Published:** 2023-04-18

**Authors:** Shengshuai Shan, Fang Liu, Edith Ford, Ruth B. Caldwell, S. Priya Narayanan, Payaningal R. Somanath

**Affiliations:** a Clinical and Experimental Therapeutics, College of Pharmacy, University of Georgia, Augusta, GA, 30912, USA; b Research Department, Charlie Norwood VA Medical Center, Augusta, GA, 30901, USA; c Vascular Biology Center, Augusta University, Augusta, GA, 30912, USA; d Culver Vision Discovery Institute, Augusta University, Augusta, GA, 30912, USA

**Keywords:** Triciribine, Proliferative retinopathy, Oxygen-induced retinopathy, Neovascular tufts, Vascular permeability, Neuroinflammation

## Abstract

Proliferative retinopathies are the leading cause of irreversible blindness in all ages, and there is a critical need to identify novel therapies. We investigated the impact of triciribine (TCBN), a tricyclic nucleoside analog and a weak Akt inhibitor, on retinal neurovascular injury, vascular permeability, and inflammation in oxygen-induced retinopathy (OIR). Post-natal day 7 (P7) mouse pups were subjected to OIR, and treated (i.p.) with TCBN or vehicle from P14-P16 and compared with age-matched, normoxic, vehicle or TCBN-treated controls. P17 retinas were processed for flat mounts, immunostaining, Western blotting, and qRT-PCR studies. Fluorescein angiography, electroretinography, and spectral domain optical coherence tomography were performed on days P21, P26, and P30, respectively. TCBN treatment significantly reduced pathological neovascularization, vaso-obliteration, and inflammation marked by reduced TNFα, IL6, MCP-1, Iba1, and F4/80 (macrophage/microglia markers) expression compared to the vehicle-treated OIR mouse retinas. Pathological expression of VEGF (vascular endothelial growth factor), and claudin-5 compromised the blood-retinal barrier integrity in the OIR retinas correlating with increased vascular permeability and neovascular tuft formation, which were blunted by TCBN treatment. Of note, there were no changes in the retinal architecture or retinal cell function in response to TCBN in the normoxia or OIR mice. We conclude that TCBN protects against pathological neovascularization, restores blood-retinal barrier homeostasis, and reduces retinal inflammation without adversely affecting the retinal structure and neuronal function in a mouse model of OIR. Our data suggest that TCBN may provide a novel therapeutic option for proliferative retinopathy.

## 1. Introduction

It has been estimated that the number of diabetics patients could double by 2060 [1], corresponding with a predicted rise in the incidence of proliferative retinopathy (PR) cases, which include diabetic retinopathy (DR) and retinopathy of prematurity (ROP) [2]. DR and ROP are significant causes of visual impairment and blindness affecting children and working-age adults in industrialized countries [3]. Pathological retinal neovascularization (RNV) associated with endothelial cell (EC) dysfunction, vascular injury, and inflammation are key pathological changes in PR [4]. Abnormal blood vessel growth is also characterized by compromised blood-retinal barrier (BRB) function, which can lead to hemorrhage, and eventually vision loss [5]. Current treatment options for PR such as laser photocoagulation [6] and intravitreal anti-vascular endothelial growth factor (VEGF) therapies have the disadvantage of causing retinal damage and long-term complications, respectively [7]. Uncertainty about the molecular changes associated with EC dysfunction remains a bottleneck in developing therapeutics for proliferative RNV for no curative therapies are currently available. These challenges highlight a critical need to characterize the underlying mechanisms and identify novel therapies that simultaneously target pathological angiogenesis and inflammation in PR.

The Akt pathway maintains healthy blood vessels [4,8-13] via EC-barrier protection [13,14]. The loss of Akt1 [12] and EC-specific loss of Akt1 [13] in mice results in vascular leakage via reduced expression of Claudins, particularly claudin-5 (Cldn5). Interestingly, reports suggest that hyperactivation of Akt to pathological levels also causes vascular malformations and increased vascular permeability in several vascular beds, including the retinal vasculature [15], suggesting that a ‘fine-tuning’ of Akt activity is necessary for healthy vasculature and that either a significant reduction or hyperactivation will likely result in vascular malformations. Akt hyperactivation has been implicated in DR and other forms of PR [16,17]. Akt is activated and Cldn5 and VE-cadherin expressions are elevated in human retinal ECs (HRECs) in response to hyperglycemia and advanced glycation end-products in vitro [18]. Literature indicates that a ‘fine-tuning’ of Akt activity is necessary for vascular homeostasis, either total inhibition or hyperactivation results in vascular abnormalities [12,13,15,19,20]. Hence, despite its upregulation in RNV, total Akt inhibition will only worsen the injury and vascular dysfunction. An agent that fine-tunes Akt activity in dysfunctional retinal ECs and suppresses inflammation will make an excellent therapy for proliferative RNV.

Triciribine (TCBN) is a tricyclic nucleoside analog of adenosine and a weak Akt inhibitor [21,22]. TCBN is phosphorylated to its active metabolite (TCBN-P) by adenosine kinase in a variety of human cells [23,24]. TCBN has shown anti-cancer properties in clinical studies, where it has exhibited excellent pharmacokinetic and safety profiles [25,26]. Studies from our laboratory have shown that treatment with TCBN offers vascular protection in various organ injury models [18,24,27,28]. TCBN enhances the resolution of lung injury by increasing the number of anti-inflammatory regulatory T cells and by inhibiting aberrant vascular leakage [28], suggesting its potential benefits in treating vascular injury and inflammation. The potential efficacy of TCBN in reducing pathological RNV in PR remains to be determined. A better understanding of TCBN on pathological RNV and inflammation may pave the way to new therapies for PR. Our study tested the efficacy of TCBN as an alternate therapy to reduce vascular complications and inflammation in PR.

We determined the impact of TCBN on RNV injury, vascular permeability, and inflammation in the mouse model of oxygen-induced retinopathy (OIR), a highly reproducible experimental model for mechanistic investigations of PR [29,30]. We hypothesized that hyperactivation of Akt and increased VEGF production in the retinas of mice with OIR would result in increased expression of Cldn5 and that this would be associated with increased vascular permeability, RNV tuft formation, inflammation, and gliosis. We further predicted that this would be reversed by TCBN treatment which would suppress the hyperactivity of Akt which would lead to decreases in VEGF expression and inhibition of inflammation.

## 2. Material and methods

### 2.1. Animals, the mouse model of OIR and TCBN treatment

Wild-type (WT) mice on a C57BL/6J background (Jackson Laboratories, Bar Harbor, ME) used for this study were maintained in our animal facility. All the animal experiments were conducted per the Association for Research in Vision and Ophthalmology (ARVO) guidelines on the use of animals in vision and ophthalmic research. The protocols used in the current study were approved by the Institutional Animal Care and Use Committee of Augusta University and Charlie Norwood VA Medical Center, Augusta. All efforts were made to reduce the number of animals used for experiments and to minimize animal suffering during experimental procedures.

Oxygen-induced retinopathy was induced in the mice as described in our previous studies [31-33]. In brief, neonatal pups from postnatal day 7 (P7), along with the nursing mothers, were exposed to 70 % oxygen in a hyperoxia chamber (BioSpherix, Parish, NY) equipped with a Cyto-centric^®^ High Infusion Rate O2 Controller (PROOX model 360, BioSpherix, Parish, NY) for 5 consecutive days and then returned to room air (RA, 21 % oxygen) on P12. Conditions such as humidity, temperature, and the health of the animals while in the chamber were assessed twice daily. Age-matched mice kept in RA and raised under identical light and temperature conditions throughout postnatal development served as the RA controls.

For pharmacological inhibition studies, the experimental (or control) mice received intraperitoneal injections (i.p.) of TCBN (1 mg/kg) [27,28] or vehicle (saline) for 3 days (P14-P16). The time was chosen to closely mimic the phase of PR. The optimum dose for TCBN was selected following preliminary studies using various doses (0.5, 1.0. 2.0, and 5.0 mg/kg body weight). Animals were euthanized under deep anesthesia, and the eyeballs or retinas were collected and processed as described below.

### 2.2. Analysis of vaso-obliteration and neovascular tuft formations on retinal flat mounts

Immunostaining of retinal flat mounts was performed as previously described [32,34]. In brief, eyes were removed, fixed in 4 % paraformaldehyde (PFA) overnight at 4 °C, and cornea, sclera, lens, vitreous, and hyaloid vessels were removed, after which four radial incisions were made to allow retinal flattening. Retinas were permeabilized with 10 % Triton X-100 for 20 min and blocked with 10 % normal goat serum containing 1 % BSA and 0.1 % Triton X-100 for 1 h at room temperature. Retinas were stained with Alexa555-labeled *Griffonia simplicifolia* Isolectin B4 (IB4, 1:100) (red, blood vessels) (Table 1) overnight at 4 °C. The next day, retinas were washed three times in PBS and flat-mounted in a mounting medium (Vectashield; Vector Laboratories, Burlingame, CA). Flatmounts were imaged using a confocal microscope (Zeiss LSM 510 META, Thornwood, NY). Assessment of the retinal avascular area and neovascular tufts were performed using the NIH ImageJ software program (Version 1.53 s, NIH, Bethesda, MD)., according to the reported method [35].

### 2.3. Retinal fluorescein angiography (FA) and OIR vascular outcome assessment

Retinal vascular permeability in vivo was evaluated according to the previously published methods [36,37]. WT mice on P21 were anesthetized by intramuscular injection of rodent anesthesia cocktail (ketamine, 73 mg/kg and xylazine hydrochloride, 7.3 mg/kg body weight, respectively). Pupils were dilated using 1 % tropicamide (Bausch and Lomb Corp., Rochester, NY). The mouse was placed on the imaging platform of the FA equipped with two MICRON IV in vivo high-resolution retinal imaging microscopes (Phoenix Technology). Hypromellose eye drops (2.5 % Goniovisc; Sigma Pharmaceuticals, LLC, Monticello, IA) were applied to keep surface moisture during the procedure. Mice were administered an injection (i.p.) of 10–20 μL of fluorescein sodium (10 %Lite; Apollo Ophthalmics, Newport Beach, CA). Rapid acquisition of fluorescent images ensued for approximately 5 min. Fluorescein leakage was assessed by comparing the fluorescence intensity from images collected after fluorescein injection. The fluorescence intensity of FA per mouse retina was calculated using the ImageJ software program after conversion to binary images and then normalized to that of normal WT mice, which was arbitrarily set at 100. Data were subjected to statistical analysis, as previously described [37].

The retinal vascular development was evaluated in the late phase of OIR mice (P30) in vivo using fluorescein angiography (FA). The late phase denotes any time point between P30 to P34 [38]. Using the MICRON IV retinal imaging system, retinal FA images with 50° fields of view were obtained in one eye of each mouse. Quantification of Retinal Vascular Features was performed using customized MATLAB software (version release R2022a, Mathworks, Natick, MA) for retinal vein width (RVW) and retinal arterial tortuosity (RAT), according to the established methods [39,40]. To avoid selection bias, the same image was used to measure vessel width and tortuosity. For RVW measurement, semi-automated calibrations were used to select two points aligned horizontally at both edges of the vein (the largest and least tortuous vessel in the image) to delineate a width measurement. For RAT measurements, three arteries were chosen and branch points were selected using a cursor, starting from the base of the optic nerve. All the image analyses were performed by the QuantBV program using MATLAB software.

### 2.4. Immunofluorescence staining on retinal sections

Immunostaining of retinal cryostat sections was performed as per the methods established in our laboratory [41-44]. Briefly, eyes were enucleated and fixed in 4 % paraformaldehyde (PFA) overnight at 4 °C, equilibrated in 30 % sucrose for 24 h, and embedded in tissue-tek optimum cutting temperature compound (Sakura Finetek, Torrance, CA). Cryostat sections (10 μm) were permeabilized in 0.1 % Triton X-100 (20 min) followed by blocking in 10 % normal goat serum containing 0.1 % Triton X-100 for 1 h at room temperature. Sections were incubated with primary antibodies (Table 1) overnight at 4 °C. Next day, the specimens were washed with 1X phosphate-buffered saline (PBS) (3 times) and then incubated with fluorescein-conjugated secondary antibodies (Table 1) for 2 h at room temperature in the dark. The anti-fade mounting medium with DAPI (Vectashield; Vector Laboratories, Burlingame, CA, USA) was applied to the sections on the cover slip after rinsing with PBS.

### 2.5. Imaging of retinal cryostat sections and quantification

Images (20X) were taken in tuft regions of the OIR retina, or the comparable region of the RA retina using a Zeiss 780 Inverted Confocal microscope (Thornwood, NY, United States). A minimum of 5 animals per group were included in each study. For quantification, a minimum of three sections (20 μm apart) per animal were utilized for each antibody. As described in our earlier studies [42,44], images were acquired at 500 μm from the optic nerve head of the retinal sections using a confocal microscope, resulting in a minimum of 6 images per mouse per antibody. Quantification of F4/80 (EGF-like module containing mucin-like hormone receptor-like 1) and Iba-1 positive cells was performed on 20X images using the “point tool” functional module of the ImageJ program. Fluorescence intensity was measured as integrated density (IntDen), and the mean values were calculated and expressed as the fluorescence intensity per field of view (FOV) for every marker used (e.g., F4/80, Iba-1, GFAP, Cldn5, and αSMA). The area of fields measured per marker was maintained uniformly throughout all groups and values were normalized relative to the percentage of the WT RA control group.

### 2.6. Western blot analysis

Retinas were collected and stored at −80 °C. For analysis of albumin leakage across the blood-retinal barrier, mice were perfused with PBS to clear blood out of the retina vessels before collection, and retinas were processed for Western blot analysis. Retinas were homogenized using a hand homogenizer in 1X RIPA lysis buffer (Millipore, Billerica, MA) containing 1x protease and phosphatase inhibitors (Thermo Scientific, Waltham, MA) and centrifuged at 12,000g, 15 min to prepare the protein extracts. Protein concentration was measured using a Pierce BCA protein assay kit (Thermo Scientific, Waltham, MA). Retinal proteins were separated on SDS-PAGE then transferred to nitrocellulose membranes (Millipore, Billerica, MA), and blocked in 5 % non-fat dry milk in 1X Tris-buffered saline (TBS) with 0.1 % Tween-20 (TBST) for 1 h. Membranes were incubated overnight at 4 °C with primary antibodies (Table 1). The next day, membranes were washed with 1X TBST (3 times) and incubated with secondary antibodies (anti-rabbit or anti-mouse HRP-conjugated secondary antibody, Table 1) for 2 h at room temperature. After further washing, the immunoreactive proteins were detected using enhanced chemiluminescence (ECL) system (Thermo Scientific, Waltham, MA) and ChemiDoc Imaging System (Bio-Rad, Hercules, CA). Data were quantified by densitometry using ImageJ software and normalized to β-actin as the loading control.

### 2.7. RNA isolation and quantitative RT-PCR

The total mouse retina was homogenized by a Micro-Tube homogenizer (SP BEL-ART) using QIAzol Lysis Reagent (Qiagen, Hilden, Germany). Total RNA from homogenized retinal tissues was extracted using miRNeasy mini kit (Qiagen). The concentration of RNA was measured using a Nanodrop Lite Spectrophotometer (Thermo Fisher Scientific, Waltham, MA, USA). Around 500 ng of total RNA was used for cDNA synthesis using a High-Capacity cDNA Reverse Transcription Kit (Applied Biosystems, Waltham, MA, USA). Quantitative PCR was carried out by StepOnePlus^™^ Real-Time PCR System (Applied Biosystems) using Power SYBR Green Master Mix (Applied Biosystems). The sequences of primers used in this study are listed in Table 2. Data were normalized to glyceraldehyde-3-phosphate dehydrogenase (GAPDH) and hypoxanthine-guanine phosphoribosyltransferase (HPRT), and the fold change between levels of different transcripts was calculated by the ΔΔCT method.

### 2.8. Analysis of retinal structure and function

Spectral Domain-Optical Coherence Tomography (SD-OCT) is a non-invasive approach used for visualizing retinal and ocular structures and assessing retinal structural abnormalities in vivo [45]. Retinal structural changes and retinal thickness were examined using SD-OCT in the late stage of OIR (P30). Briefly, mice were anesthetized with 73 mg/kg ketamine hydrochloride and 7.3 mg/kg xylazine hydrochloride intraperitoneally. Pupils were dilated with 1 % tropicamide (Akorn, Lake Forest, IL, USA) followed by the application of GENTEAL^®^ tears lubricant eye gel (Alcon, Fort Worth, TX). To keep the cornea moist during the procedure, Systane lubricant eye drops (Alcon) were applied. B-scan images were taken using the Bioptigen Spectral Domain Ophthalmic Imaging System (SDOIS) (Bioptigen Envisu R2200, Morrisville, NC) as we previously described [44,46]. The thickness of retinal layers was generated by the DIVERS software program integrated with the imaging system.

To determine the effect of TCBN treatment on retinal function, we performed an electroretinogram (ERG) analysis of RA control and OIR mice that had been previously treated with TCBN or vehicle using the standardized technique in our core facility [47]. Briefly, ERGs were performed at P26 using the Touch/Touch feature of the Celeris Ophthalmic Electrophysiology System (Diagnosys, Lowell, MA) available at the Augusta University Vision Core. For scotopic ERGs, mice were dark-adapted for approximately 16 h before the experiment, then tested using a series of light flashes of increasing energy (0.001, 0.005, 0.01, 0.1, 0.5, and 1.0 cd s/ m2).

### 2.9. Statistical analysis

Data are presented as mean ± SD or SEM. GraphPad Prism 9 (GraphPad Software Inc., La Jolla, CA, USA) was used for statistical analysis. Data were analyzed by one-way ANOVA followed by the Tukey test for multiple comparisons. The Student’s *t*-test was used in the case of a single comparison with the means between two groups. To address the selection bias, analyses were performed in a blinded fashion and quantified using ImageJ software by three separate observers. Significance was set at a *P* value of less than 0.05.

## 3. Results

### 3.1. TCBN suppresses vaso-obliteration and pathological neovascularization in the OIR retina

**Fig. 1.**
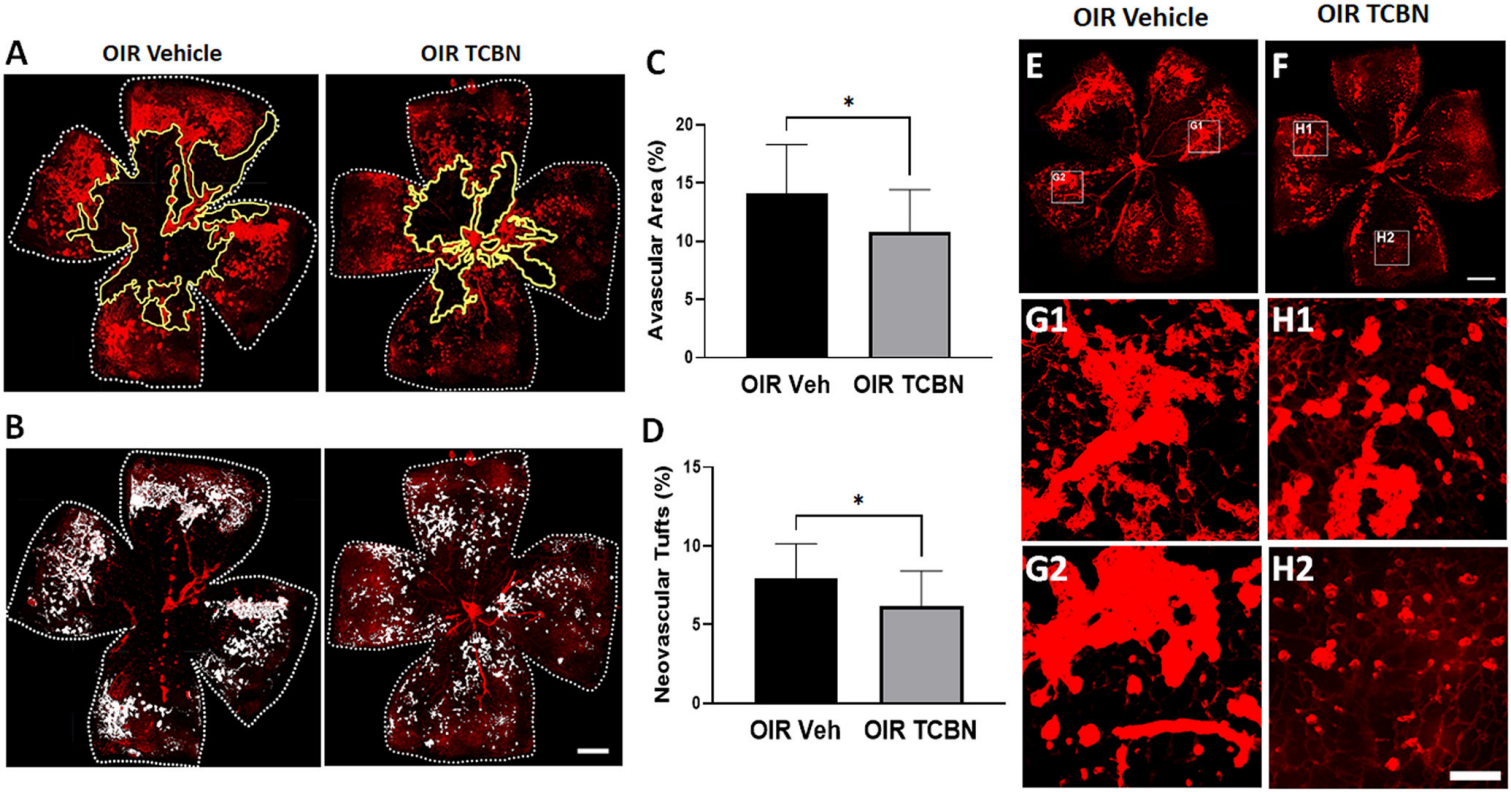
TCBN suppresses vaso-obliteration and pathological neovascularization in the OIR retina. (A-B) Representative confocal images of flat-mounted retinas (harvested on P17) stained with isolectin B4 (red, blood vessels) demonstrating avascular areas (the yellow outlined region represents the area of vaso-obliteration) and pathological angiogenesis (the white areas indicate areas of neovascular tufts). (C) The retinal avascular area was quantified using NIH ImageJ software. Assessment of vaso-obliteration is expressed as the central avascular areas divided by the total area of the retina. (D) Quantification analysis of the total area of neurovascular tufts was calculated as the percentage of the retinal neovascular tuft area, measured using the semi-automated thresholding technique in the NIH ImageJ. (E-F) Representative flat-mount images demonstrating changes in pathological neurovascular tufts in both OIR vehicle- and OIR TCBN-treated retinas, with two insets (boxed areas). G1 and G2 show representative neurovascular tuft areas from vehicle OIR retinas while H1 and H2 show representative neurovascular tuft areas from TCBN-treated OIR retinas. Data are presented as mean ± SD. *p < 0.05 versus OIR vehicle, n = 10–12 per group. Scale bar 500 μm.

To evaluate the effect of TCBN on OIR-induced vascular injury, analyses of vaso-obliteration and neovascular tufts were performed on P17 flat-mounted OIR retinas stained with isolectin B4. Fig. 1A and B demonstrate the changes in vaso-obliteration (area outlined in yellow), and neovascularization (presented as white areas) in the vehicle and TCBN-treated OIR retinas. Reductions in vaso-obliteration and neovascularization are observed in the TCBN-treated OIR retinas, compared to the vehicle-treated OIR retinas (Fig. 1A and B). Quantitative analysis revealed a significant decrease in vaso-obliteration by 22.9 % (p < 0.05) (Fig. 1C) and pathological neovascularization by 21.8 % in response to TCBN treatment (p < 0.05) (Fig. 1D). Flatmount images demonstrated changes in the relative sizes of the neurovascular tufts in vehicle- and TCBN-treated OIR retinas (Fig. 1E and F). The neovascular tufts in TCBN-treated OIR retinas (Fig. 1F, H1, and H2), appeared smaller compared to the vehicle-treated OIR retinas (Fig. 1E, G1, and G2). This reduction in neovascular tufts indicates the beneficial effect of TCBN treatment in blocking pathological neovascularization. The decrease in the avascular area suggests that the TCBN treatment also promotes physiological vascular repair.

### 3.2. TCBN treatment decreased OIR-induced vascular permeability

**Fig. 2.**
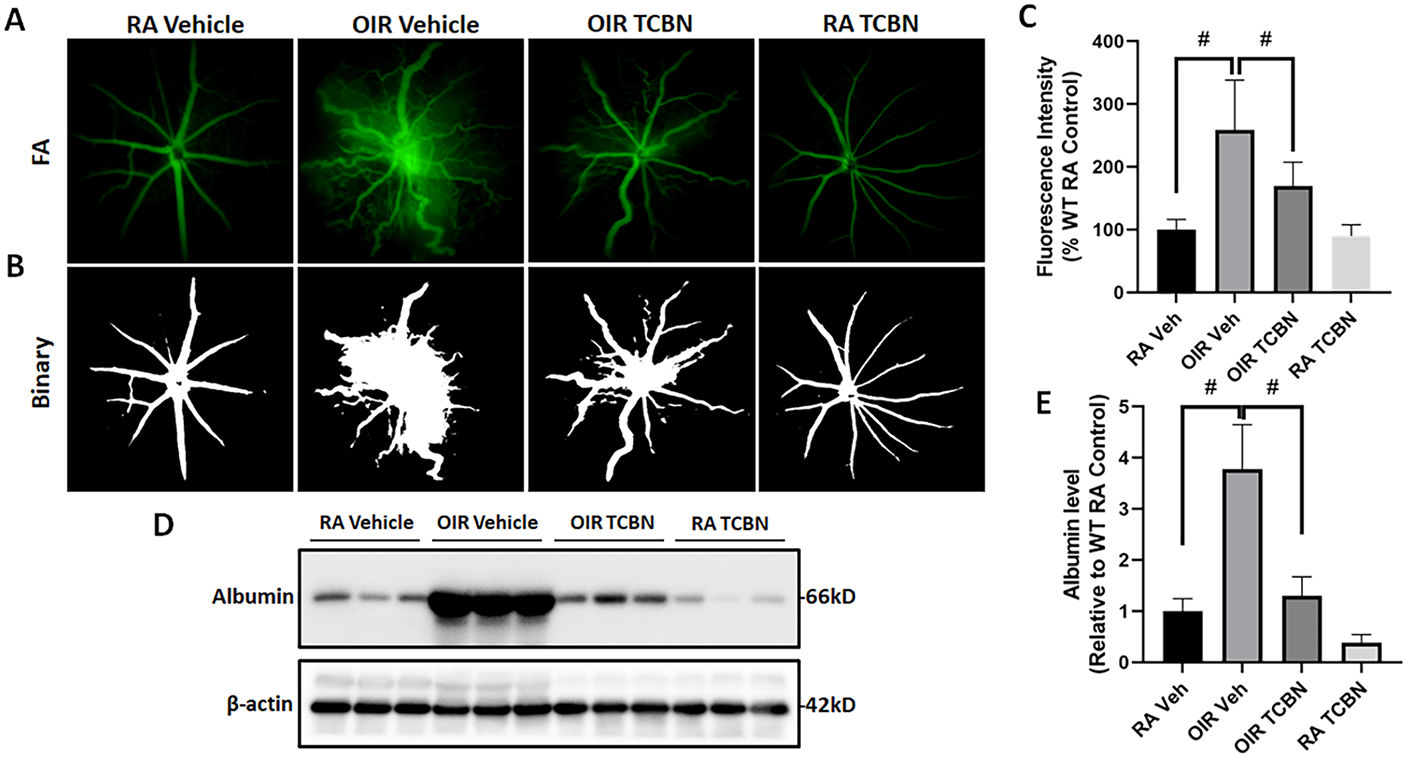
TCBN treatment decreases vascular permeability in the OIR retina. (A-B) Fluorescein angiography (FA) analysis (performed at P21) of RA and OIR mice treated with vehicle or TCBN (P14-P17). Data presented are representative pictures taken at a constant interval of every mouse studied in each group, along with respective binary images. (C) The fluorescence intensity per mouse retina was calculated by the NIH ImageJ software. (D) Western blot analysis showing the extravasated retinal albumin in RA and OIR mice treated with vehicle or TCBN. (E) Quantification of albumin protein expression levels relative to the β-actin, compared with RA control, which was arbitrarily set at 1.0. Data are presented as mean ± SD. #p < 0.001; n = 6–14 per group for FA analysis. n = 6 per group for Western blot analysis.

The effect of TCBN treatment on retinal vascular permeability in OIR-induced vasculopathy was determined by analysis of fluorescein leakage using FA and albumin extravasation using Western blotting of retinal lysates. The FA analysis of the vehicle- (Fig. 2A and B) and TCBN-treated (Fig. 2A and C) RA mouse retinas showed normal vessel-filling patterns with no observable fluorescein leakage into the surrounding tissue (89.89 ± 17.95 vs. 100.0 ± 16.25, p > 0.05). In contrast, vehicle-treated OIR retinas exhibited extensive fluorescein leakage compared to RA controls (259.0 ± 79.08 vs. 100.0 ± 16.25, p < 0.001) (Fig. 2A and C). This leakage was significantly inhibited by TCBN treatment (259.0 ± 79.08 vs. 169.7 ± 37.70, p < 0.001) (Fig. 2 C). Further, the western blot analysis of albumin levels in the perfused P17 retinas revealed a significant increase in the extravasated albumin in the OIR retinas compared with the RA controls (p < 0.001) (Fig. 2D and E). Of note, the albumin extravasation was significantly reduced in the TCBN-treated OIR retinas as compared with the vehicle-treated OIR group (1.297 ± 0.377 vs. 3.782 ± 0.867, p < 0.001) (Fig. 2D, E). Although we observed a modest decrease in albumin extravasation in the TCBN-treated RA group as compared with the vehicle-treated RA group (Fig. 2D), the difference was not statistically significant (p > 0.05) (Fig. 2E). Collectively, these results show the efficacy of TCBN in preventing OIR-induced increase in retinal vascular permeability.

### 3.3. TCBN inhibits the OIR-induced increase in VEGF and Cldn5 expression, and retinal vascular remodeling

**Fig. 3.**
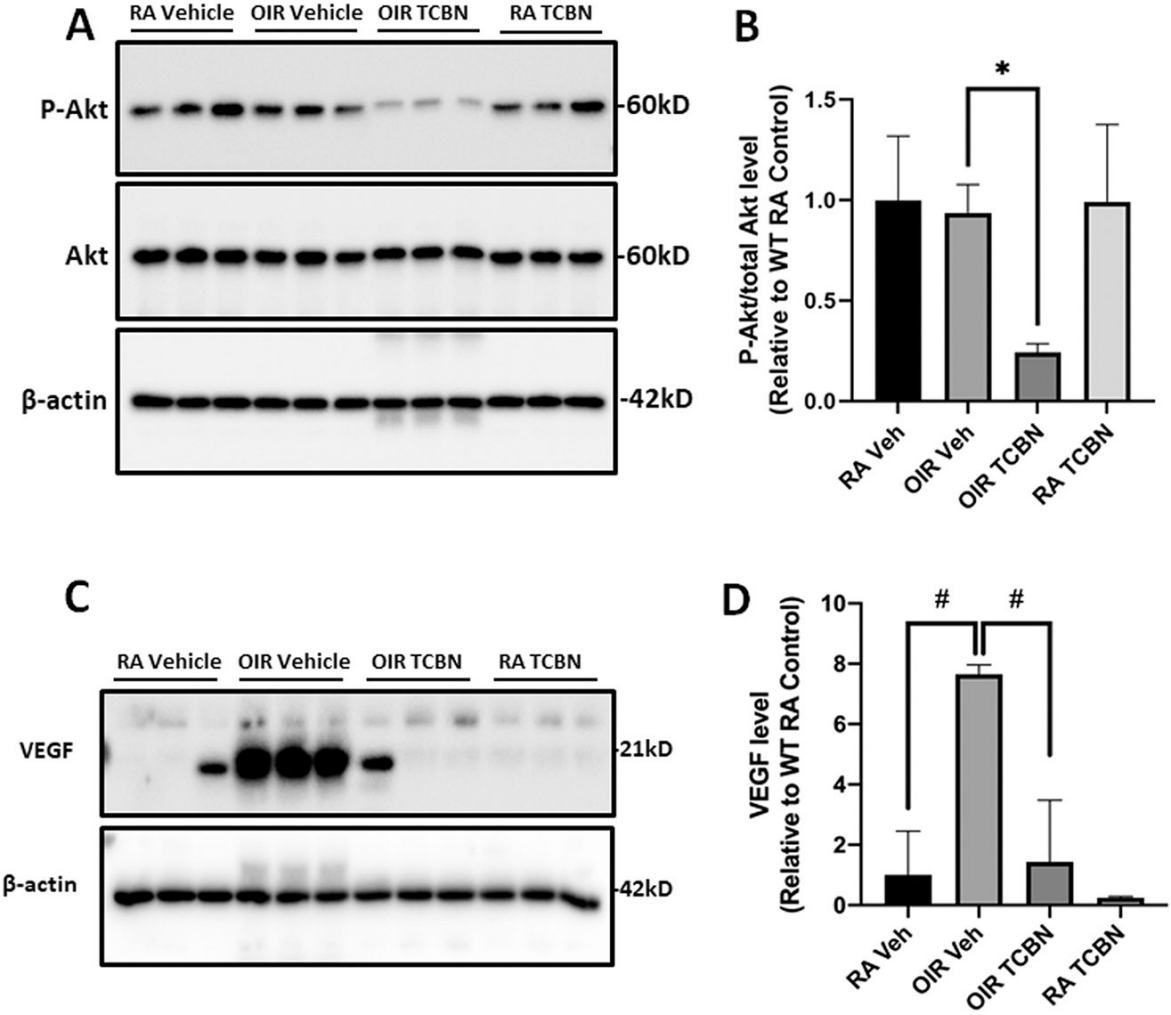
TCBN treatment reduces Akt phosphorylation and VEGF expression in the OIR retina. (A) Representative Western blot images of retinal lysates (RA Vehicle, RA control, OIR, and OIR + TCBN) probed with phosphorylated Akt (Ser473), pan Akt, and β-actin. (B) Bar graph showing quantification of phosphorylated Akt expression (band densitometry) analyzed using NIH ImageJ software. (C) Representative Western blot images of retinal lysates (RA Vehicle, RA control, OIR, and OIR + TCBN) probed with VEGF and β-actin. (B) Bar graph showing quantification of VEGF expression (band densitometry) analyzed using NIH ImageJ software. Data are presented as mean ± SD. #p < 0.001; *p < 0.05. n = 3 per group.

We determined the changes in the expression of phosphorylated Akt (pSer473 Akt) and VEGF, the primary mediator of vascular permeability and vascular complications in PR. Although pSer473 Akt upregulation was not evident in the OIR retinas compared to RA controls (likely because Akt activation could be an early event), there was a significant reduction in pSer473 Akt levels with TCBN treatment (0.242 ± 0.043 vs. 0.937 ± 0.140, p < 0.05) (Fig. 3A and B). Our data also revealed a robust increase in VEGF in the OIR retina compared to RA controls (7.652 ± 0.307 vs. 1.000 ± 1.452, p < 0.001), which was significantly inhibited by TCBN treatment (1.441 ± 2.039 vs. 7.652 ± 0.307, p < 0.001) (Fig. 3C and D).

**Fig. 4.**
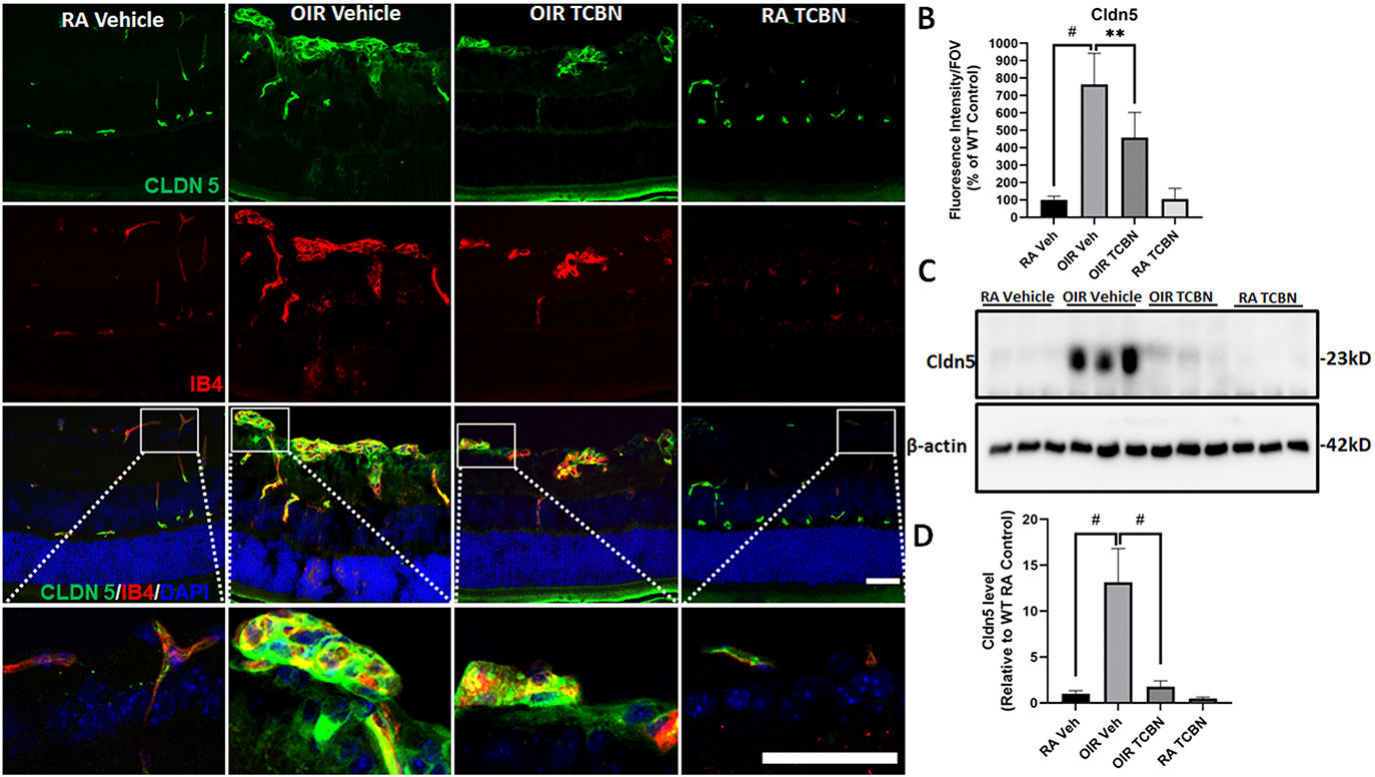
Upregulation of claudin-5 (Cldn5) expression in the OIR retinal tufts is blunted by TCBN treatment. (A) Representative confocal images of RA and OIR retinas (with vehicle or TCBN treatment) immunostained with Cldn5 and IB4 showing markedly increased expression of Cldn5 in the tuft areas in the OIR retinas, compared with RA control and its suppression by TCBN administration. (B) Histogram presenting the quantification of Cldn5 expression measured as fluorescent intensity per field of view (FOV) used. (C) Western blot analysis showing the retinal Cldn5 expression in RA and OIR mice treated with vehicle or TCBN. (D) Quantification of Cldn5 protein expression levels relative to the β-actin, compared with RA control, which was arbitrarily set at 1.0. GCL, ganglion cell layer; IPL, inner plexiform layer; INL, inner nuclear layer; OPL, outer plexiform layer; ONL, outer nuclear layer. Data are presented as mean ± SD. #p < 0.001; * *p < 0.01; *p < 0.05. n = 5 per group for immunostaining analysis. n = 3 per group for Western blot analysis. Scale bar 50 μm.

We next examined the expression of Cldn5, a major TJ protein, in vascular structures double labeled with IB4. The immunofluorescence analysis revealed Cldn5 staining (green) colocalizing with IB4 (red) (Fig. 4A). Fluorescence intensity analysis showed a significant increase in Cldn5 expression in the RNV tufts of the vehicle-treated OIR mice. In contrast, TCBN-treated OIR retinas showed a significant decrease in the Cldn5 immunoreactivity, in the blood vessels compared with the vehicle-treated OIR retinas (~40 %) (572.6 ± 181.2 vs. 955.9 ± 289.5, p < 0.01) (Fig. 4A and B). Immunoblotting studies further confirmed the significant upregulation in Cldn5 expression in the retinas of vehicle-treated OIR mice, which was normalized by TCBN treatment with a >90 % reduction in Cldn5 expression (Fig. 4C and D). These results, combined with our previous report in HRECs in vitro [18], suggest that upregulation of Cldn5 expression could be pathological in PR and that TCBN treatment can prevent it.

**Fig. 5.**
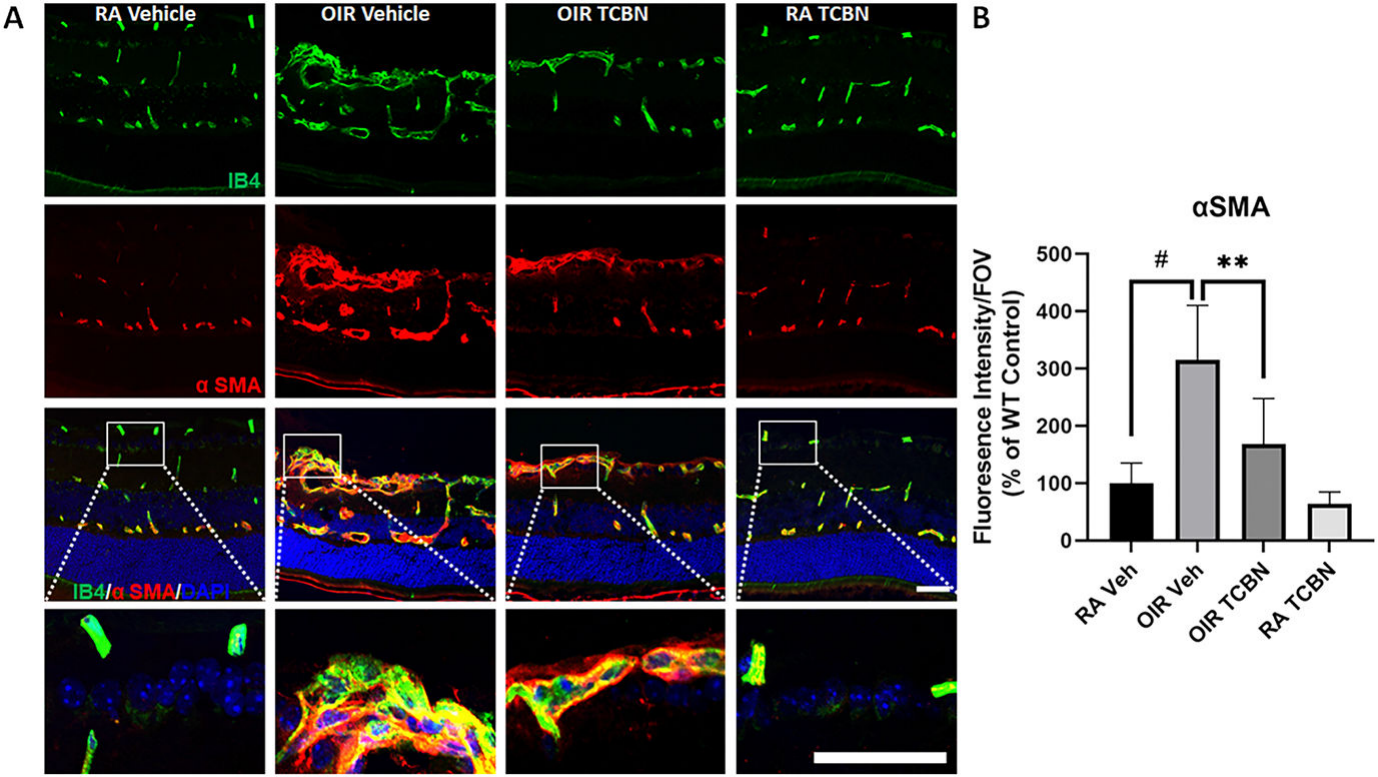
TCBN attenuates pathological vascular remodeling in the OIR retina. (A) Representative confocal images of RA and OIR retinas showed a marked increase in αSMA expression in the tuft areas of the OIR retinas, which was blunted by TCBN treatment. (B) Histogram representing the quantification of the αSMA-covered blood vessel area. Fluorescence intensity was measured by the NIH ImageJ program and the mean values were calculated and expressed as the fluorescence intensity per field of view (FOV). Data are presented as mean ± SD. #p < 0.001; * *p < 0.01; n = 5 per group. Scale bar 50 μm.

To investigate the effect of TCBN on pathological vascular remodeling in the OIR retina, we immunostained retinal sections with antibodies against αSMA (smooth muscle cell actin-α), a marker of vascular pathology and retinal fibrosis [48], and IB4. Our analysis revealed a significant increase in αSMA expression in the neovascular tufts in the OIR retinas, which was reduced by −50 % by TCBN treatment (168.7 ± 79.06 vs. 315.1 ± 94.89, p < 0.01) (Fig. 5A and B). In addition, there were no significant changes in αSMA expression between the RA vehicle group compared to the RA-TCBN group. Our results indicated that TCBN treatment attenuates pathological vasculature remodeling in the OIR retina.

### 3.4. TCBN treatment augments retinal vascular recovery

**Fig. 6.**
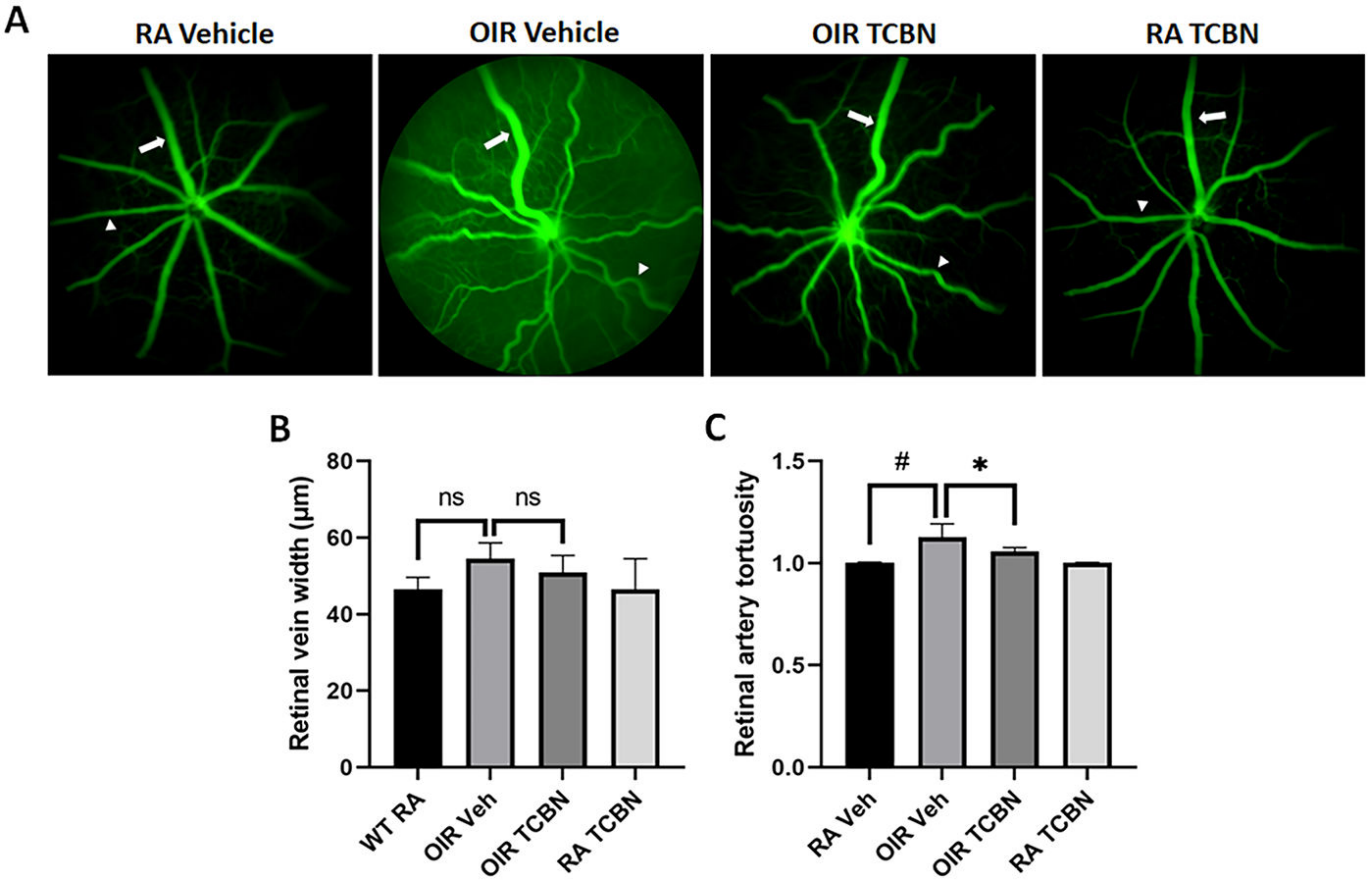
TCBN treatment augments retinal vascular recovery in the late stage of OIR. (A) Representative FA retinal images showing changes in the vascular features such as retinal vein width (RVW) and retinal arterial tortuosity (RAT) on P30 by fluorescein angiography (FA) in the RA vehicle, OIR vehicle, TCBN OIR, and RA TCBN treatment groups. (B) Quantification of the RVW, performed using Matlab software demonstrates no difference among groups. (C) Analysis of the RAT index performed using Matlab software showing changes in OIR retinas and the impact of TCBN treatment. Arrows indicate representative vessel used for RVW and arrowheads represents those used for RAT analysis. Data are presented as mean ± SD. #p < 0.001; *p < 0.05; ns, No significance. n = 4–7 per group.

Retinal vein width (RVW) and arterial tortuosity (RAT) are reliable assessment markers of vasculopathy in OIR [39]. To explore the potential effect of TCBN on retinal vascular recovery, we did a FA analysis of the mouse OIR retinas at P30. Vehicle-treated RA mouse retinas exhibited no vessel tortuosity at P30. In contrast, the retinas of mice in the vehicle-treated OIR group exhibited abnormalities, including venous dilatation and obvious arterial tortuosity (arrows indicate veins and arrowheads indicate arteries) (Fig. 6A), which was reduced by TCBN treatment. The quantitative analysis, however, did not show a statistical difference in the retinal vein width (RVW) in TCBN-treated versus the vehicle-treated OIR retinas (50.98 ± 4.370 μm vs. 54.57 ± 4.065 μm, p > 0.05) (Fig. 6B). The TCBN-treated OIR retina showed less tortuous arteries than the vehicle-treated OIR retina (1.058 ± 0.019 vs. 1.127 ± 0.066, p < 0.05) (Fig. 6C). There was no significant difference in RVW index between the RA vehicle group and RA TCBN group (46.54 ± 3.106 μm vs. 46.53 ± 7.959 μm, p < 0.05) (Fig. 6B). The RAT index did not differ between the two RA groups (Fig. 6C). Our results demonstrated that TCBN-treatment improves retinal vascular recovery in mice following the OIR treatment.

### 3.5. Elevated pro-inflammatory cytokines and chemokines in the OIR retinas are suppressed by TCBN

**Fig. 7.**
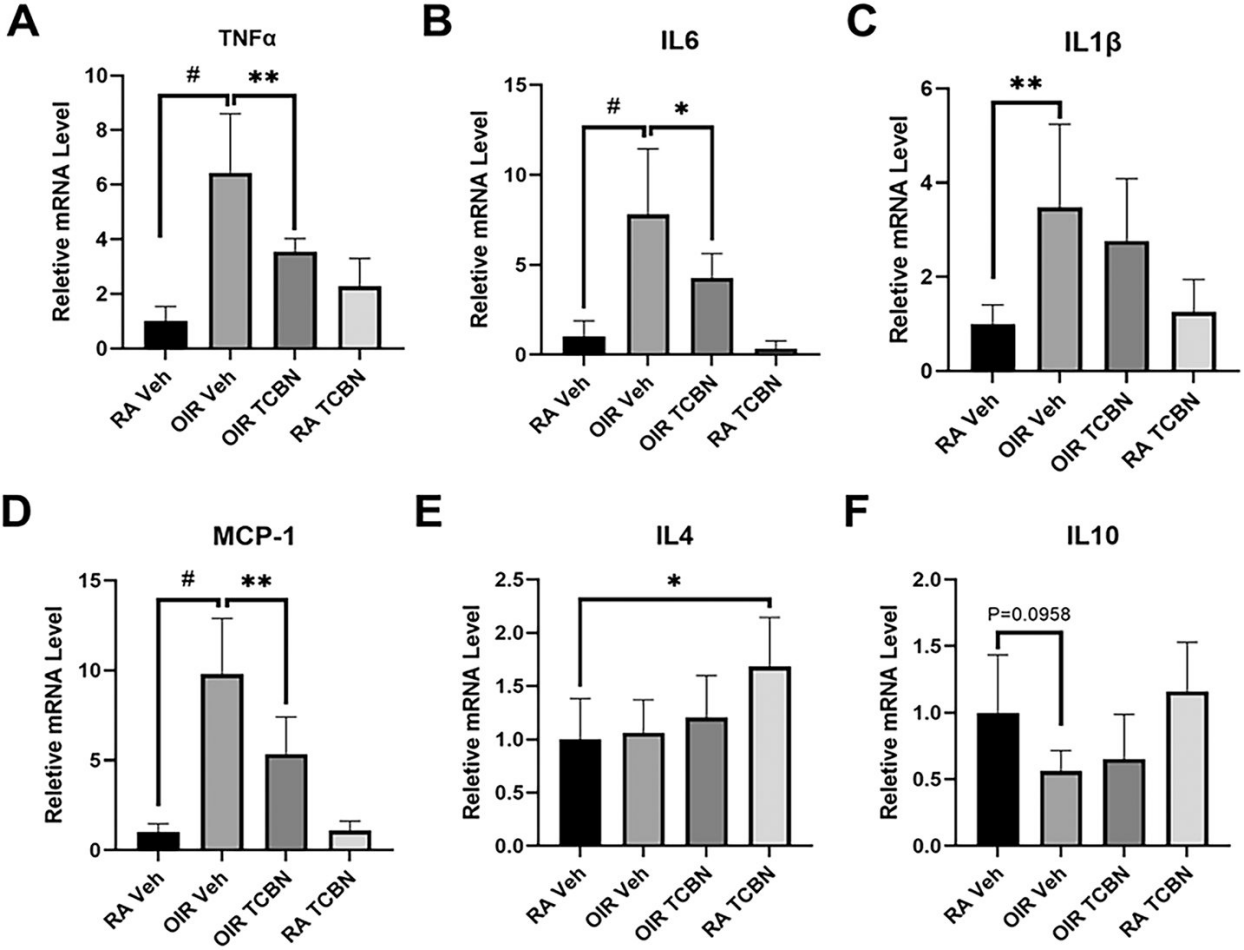
Elevated pro-inflammatory cytokines and chemokines in the OIR retinas were suppressed by TCBN. (A-D) Quantitative RT-PCR analysis demonstrating changes in the mRNA levels of pro-inflammatory cytokines and chemokines IL-1β, TNFα, IL-6, and MCP-1, respectively in the retinal samples from RA and OIR mice treated with vehicle or TCBN, normalized to GAPDH. (E-F) Quantitative RT-PCR analysis demonstrating changes in the mRNA levels of anti-inflammatory cytokines IL-4 and IL-10, respectively in the above samples (normalized to GAPDH). Data are presented as mean ± SD. #p < 0.001; **p < 0.01; * p < 0.05. n = 5–8 per group.

A qRT-PCR analysis of P17 OIR retinas revealed significant increases in mRNA expression of the pro-inflammatory cytokines IL-1β, IL-6, TNFα, and MCP-1 compared to the vehicle-treated RA controls (Fig. 7A-D), which suggests enhanced inflammation. Whereas OIR-induced mRNA expression of TNFα, IL-6, and MCP-1 were significantly decreased in the TCBN-treated OIR retinas as compared with vehicle-treated OIR retinas (3.553 ± 0.473 vs. 6.437 ± 2.165, p < 0.01; 4.268 ± 1.347 vs. 7.802 ± 3.642, p < 0.05; 4.255 ± 2.094 vs. 7.815 ± 3.521, p < 0.05, respectively) (Fig. 7A, B, and D), a marginal decrease in IL-1β levels with TCBN-treatment was not statistically significant (2.765 ± 1.321 vs. 3.475 ± 1.768, p > 0.05) (Fig. 7C). Interestingly, TCBN-treated OIR retinas exhibited a trend towards upregulation of anti-inflammatory genes IL4 and IL10, compared to vehicle-treated OIR retinas, however, the data did not reach statistical significance (1.255 ± 0.319 vs. 0.935 ± 0.238, p > 0.05; 0.862 ± 0.403 vs. 0.623 ± 0.163, p > 0.05, respectively) (Fig. 7E and F). Our results indicate that elevated pro-inflammatory cytokines and chemokines in the OIR retinas can be suppressed by TCBN treatment, suggesting TCBN is a promising drug to treat inflammation in PR. Whereas the presented data are normalized to GAPDH, changes in the expression of pro-inflammatory cytokines normalized to HPRT are shown in Supplemental Fig. S1.

### 3.6. TCBN treatment attenuates OIR-induced upregulation of inflammatory microglia/macrophages in the retina

**Fig. 8.**
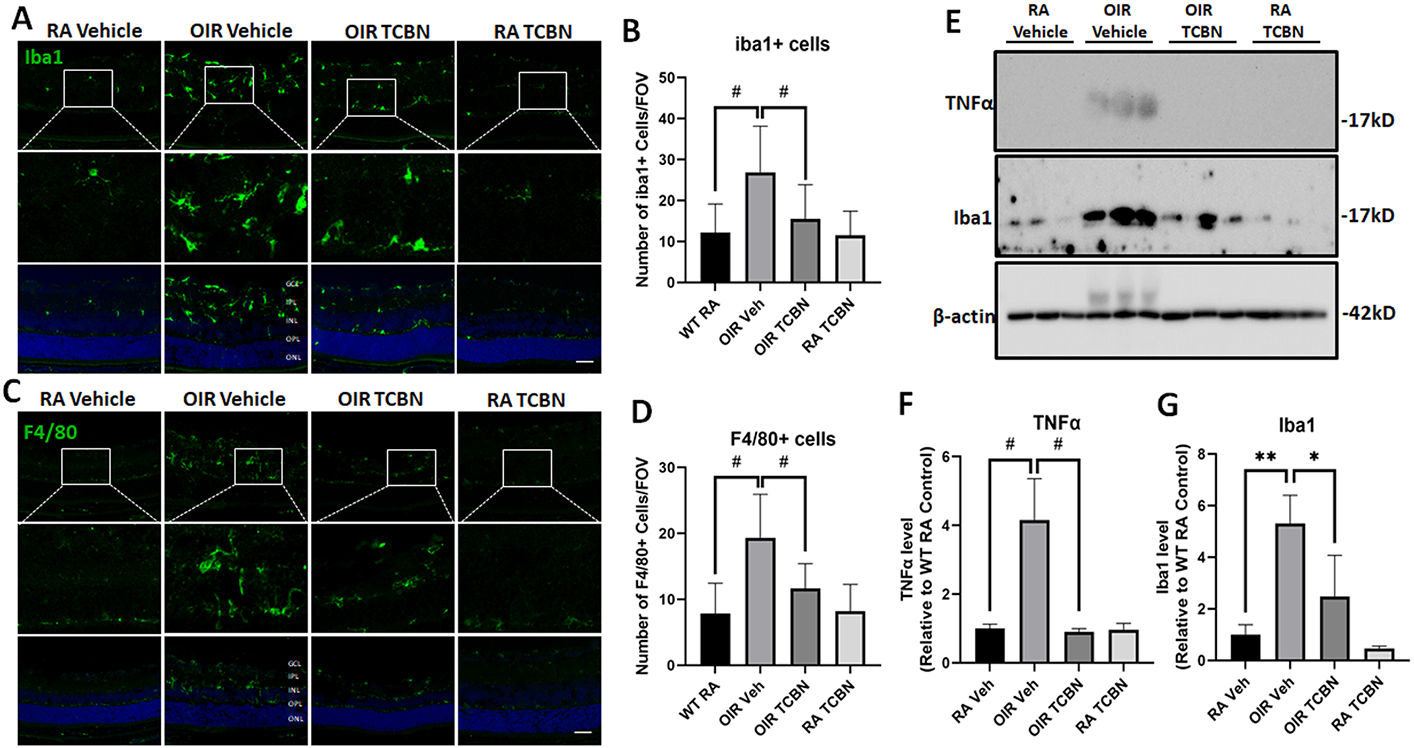
TCBN treatment reduces the number of microglia/macrophage cells in the OIR retina. (A) Representative immunofluorescence images of Iba1-labeled sections from RA and OIR retinas treated with vehicle or TCBN indicate the increased Iba1 positive cells and Iba1 expression level in the OIR retinas and its reversal by TCBN treatment. (B) Bar graph showing quantification of Iba1 positive cells and Iba1 expression level per FOV in RA and OIR groups treated with vehicle or TCBN. (C) Representative immunofluorescent images of F4/80-labeled sections from RA and OIR retinas treated with vehicle or TCBN indicate the increased F4/80 positive cells and F4/80 expression level in the OIR retinas, which were rescued by TCBN treatment. (D) Bar graph showing quantification of changes in F4/80 positive cells and F4/80 expression level per/FOV in tuft regions of the OIR retina and the impact of TCBN treatment. GCL, ganglion cell layer; IPL, inner plexiform layer; INL, inner nuclear layer; OPL, outer plexiform layer; ONL, outer nuclear layer. Nuclei were counter-stained with DAPI (blue). (E) Representative Western blot images of retinal lysates probed with TNFα and Iba1 antibodies, along with the loading control, β-actin. (F-G) Bar graph showing quantification of TNFα and Iba1 expression (band densitometry), respectively, analyzed using NIH ImageJ software. Data are presented as mean ± SD. #p < 0.001; * *p < 0.01; *p < 0.05. n = 5 per group for immunostaining analysis. n = 3 per group for Western blot analysis. Scale bar 50 μm.

Next, we investigated the impact of TCBN treatment on inflammatory cells in the OIR retina. Immunostaining of RA and OIR P17 retinal sections with Iba1 and F4/80 antibodies was performed to examine the effect of TCBN on OIR-induced changes in microglia/ macrophage (Fig. 8A and C). Our analysis revealed an increase in the number of Iba1 positive cells per field of view (FOV) in the vehicle-treated OIR retinas compared with the RA controls (1.255 ± 0.319 vs. 0.935 ± 0.238, p > 0.05; 0.862 ± 0.403 vs. 0.623 ± 0.163, p > 0.05, respectively) (Fig. 8A and B). Consistently, there was also a significant increase number of F4/80 positive cells per field of view (FOV) in the vehicle-treated OIR retinas compared with the RA controls (F4/80 + cells/FOV, 19.30 ± 2.623 vs. 7.667 ± 1.983, p < 0.001) (Fig. 8 C and D). Treatment with TCBN significantly reduced the OIR-induced increases in Iba1 and F4/80 positive cells (25.93 ± 5.234 vs. 12.73 ± 4.204, p < 0.001 and 19.30 ± 2.623 vs. 11.44 ± 2.161, p < 0.001, respectively) (Fig. 8A and C). TCBN-treated RA retinas did not show any noticeable changes in the number of Iba1 or F4/80 positive cells as compared with the vehicle-treated RA controls (Fig. 8B and D). The data from immunostaining analysis was confirmed by Western blotting, which showed a significant increase in TNFα (Fig. 8E and F) and Iba1 (Fig. 8E and G) expression in the OIR retinas compared to RA controls (4.158 ± 1.200 vs. 1.000 ± 0.126, p < 0.001; 5.307 ± 1.097 vs. 1.000 ± 0.386, p < 0.01, respectively) and its significant suppression by TCBN (p < 0.001 and p < 0.05, respectively). Our analysis indicated the potential benefits of TCBN in preventing the upregulation of retinal microglia and macrophages in response to OIR.

### 3.7. TCBN treatment reduces OIR-induced glial activation in the retina

**Fig. 9.**
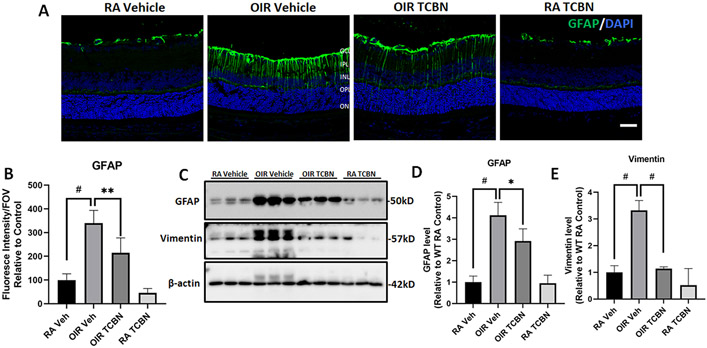
TCBN treatment inhibits glial activation in the OIR retina. (A) Representative confocal images of retinal cryosections (P17) probed with GFAP antibodies. (B) Bar graph showing quantification of GFAP fluorescence intensity performed using NIH ImageJ software. Results are presented as percentage changes compared to the WT RA control. Nuclei were counter-stained with DAPI (blue). GCL, ganglion cell layer; IPL, inner plexiform layer; INL, inner nuclear layer; OPL, outer plexiform layer; ONL, outer nuclear layer. (C) Representative Western blot images of retinal lysates probed with GFAP and vimentin antibodies. (D-E) Bar graph showing quantification of GFAP and vimentin expression (band densitometry), respectively, analyzed using NIH ImageJ software. Data are presented as mean ± SD. #p < 0.001; * *p < 0.01; *p < 0.05. n = 5–8 per group for fluorescence intensity analysis. n = 3 per group for Western blot analysis. Scale bar 50 μm.

Glial activation is a common feature in the OIR retina, which is characterized by glial fibrillary acidic protein (GFAP) upregulation in the Müller cells [49]. Our analysis showed a marked increase in the levels of GFAP in the vehicle-treated OIR retina compared to the vehicle-treated RA retina (Fig. 9A). GFAP immunoreactivity extended from the GCL through the ONL in some areas, indicating activation of the Müller cells in the OIR retinas. Quantitative analysis showed significant upregulation of GFAP in OIR retinas in comparison with the RA controls, which was significantly reduced by TCBN treatment (339.6 ± 53.91 vs. 214.3 ± 63.56, p < 0.01) (Fig. 9B). Western blot analysis revealed the upregulation of GFAP (Fig. 9C and D) and Vimentin (Fig. 9C and E) in the OIR retinas compared to RA controls (4.115 ± 0.603 vs. 1.000 ± 0.282, p < 0.001; 3.318 ± 0.371 vs. 1.000 ± 0.249, p < 0.001, respectively), and its significant suppression by TCBN treatment (p < 0.05 and p < 0.001, respectively), suggesting the ability of TCBN to suppress glial injury in the OIR retina.

### 3.8. TCBN treatment does not affect the retinal architecture

**Fig. 10.**
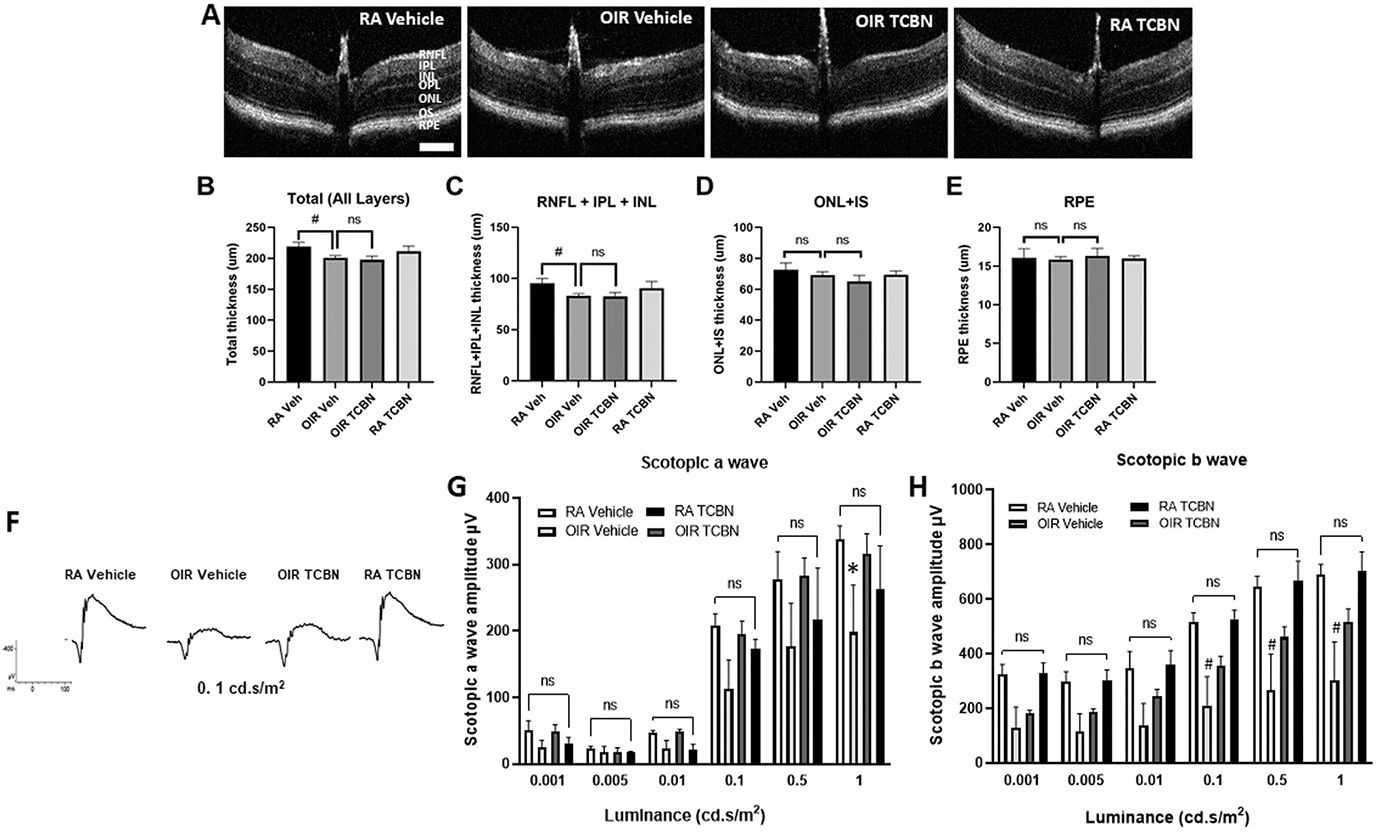
TCBN treatment does not affect the retinal architecture and function. (A) Representative images (B-scan) were obtained using the spectral domain optical coherence tomography (SD-OCT) on P30 from RA Veh, OIR Veh, TCBN OIR, and RA TCBN treatment groups. (B-E) Quantification of the total retinal thickness (from NFL to outer segment/RPE interface), and thickness for layers of RNFL+IPL+INL, ONL+IS, and RPE are presented. GCL, ganglion cell layer; IPL, inner plexiform layer; INL, inner nuclear layer; OPL, outer plexiform layer; ONL, outer nuclear layer; IS, inner segment; RPE, retinal pigment epithelium. Data are presented as mean ± SD. #p < 0.001; * *p < 0.01; *p < 0.05; ns, No significance. n = 5–8 per group. Scale bar 200 μm. (F-H) Retinal function by dark-adapted (scotopic) ERG in P26 RA control, RA TCBN, OIR, and OIR+TCBN treated mice were assessed. (F) Representative scotopic ERG responses at five stimulus contrasts (G) Scotopic a-wave amplitudes and (H) b-wave amplitudes for all four mouse groups plotted at the five light intensities. Changes in scotopic a wave and b wave amplitudes were studied at flash intensities ranging from 0.001 to 1.0 cd-seconds per meter squared (cd.s/m2). There were no significant differences between groups for rod or cone responses in P26 RA control and RA TCBN-treated mice. Data are shown as mean ± SEM. n = 3–6 per group.

Next, we used the SD-OCT to examine the impact of TCBN on the retinal structural changes in live mice on P30. In line with published studies [50], OIR mice exhibited retinal thinning as compared to the RA control (Fig. 10A). The thinning was pronounced in total retinal layers and the Ganglion Cell Complex (GCC, measured as RNFL+IPL+INL thickness) in the OIR vehicle retinas, compared to the RA vehicle retinas (201.0 ± 3.440 μm vs. 218.9 ± 7.223 μm, p < 0.001; 83.01 ± 2.293 μm vs. 95.33 ± 4.649 μm, p < 0.001, respectively) (Fig. 10B-E). No significant changes in retinal thickness were observed in the ONL+IS, and RPE layers in OIR retinas compared to RA controls (Fig. 10D and E). No significant difference was observed between the TCBN and vehicle-treated groups on the thickness of retinal layers under OIR conditions. The SD-OCT analysis of retinas on P30 showed no evidence of TCBN affecting retinal architecture, and all layers of the retina looked comparable to vehicle-treated eyes (Fig. 10), indicating that TCBN did not induce adverse reactions in the retinal neurons.

In the next step, we performed ERG analysis to determine changes in retinal function in RA control and OIR mice in the presence and absence of TCBN treatment. The scotopic and photopic ERG tests were performed on P26. While no noticeable changes were observed in photopic responses from RA and OIR groups (Supplemental Fig. S2), alterations in scotopic responses were evident in OIR mice compared to RA (Fig. 10F-H). The scotopic b amplitudes were significantly reduced at multiple intensities (0.1, 0.5, and 1 cd/s/m^2^) in the vehicle-OIR mice. Compared to WT RA controls, the OIR retinas did not show any significant changes in scotopic amplitudes (except at higher intensity, 1.0 cd.s/m2). Interestingly, treatment with TCBN demonstrated an improvement in both a and b scotopic amplitudes in OIR retinas, however, the results were not statistically significant. Responses from mice in the RA vehicle and those who received TCBN treatment were similar in both a and b scotopic measures. Overall, these results indicate that TCBN treatment does not cause any adverse effect on neurons, while it could offer neuroprotection.

## 4. Discussion

In the current study, we demonstrated that TCBN treatment during the late stage of OIR significantly reduced RNV tuft formation, and vascular leakage, accompanied by a reduction in OIR-induced increase in VEGF, Cldn5, αSMA, GFAP, and Vimentin protein expression in the retina along with increased mRNA expression of inflammatory cytokines and increased numbers of inflammatory macrophage/microglial cells. Our analyses of retinal structure and function using optical coherence tomography and ERG, respectively, showed that the TCBN treatment improved retinal function in the OIR retina without altering the retinal structure. Our results thus indicate the potential benefits of TCBN in inhibiting pathological RNV, aberrant BRB breakdown, and retinal inflammation without compromising the retinal structure and function by normalizing the pathologically elevated Cldn5 expression in the OIR retina.

The lack of understanding of the precise molecular mechanisms of PR hinders the development of efficacious and sustainable therapies for its treatment. Pharmacological agents that show promise of short-term relief in experimental models often end up developing long-term complications, primarily due to the multi-faceted nature of the PR pathology and because the compounds inhibit signaling pathways beyond their basal activity that is required for normal retinal function. Agents that will limit vascular injury and prevent inflammation in the injured retina by fine-tuning the deregulated signaling pathways will make effective therapeutics for PR. There is increasing evidence for hyperactivation of the Akt pathway in PR [17,51], deregulated Akt-mTOR pathway in diabetics [16,52], and OIR retinas [17,53] associated with increased production of growth factors such as VEGF [16]. Although this may suggest that pharmacologically targeting the Akt-mTOR pathway could be utilized to treat PR patients, several challenges remain. Targeting Akt has the disadvantage that such treatment might compromise the survival of retinal neuronal cells [54]. Additionally, it is not clear how Akt inhibition would affect the BRB in that Akt function is essential for vascular barrier function [9,11-13]. Another concern is that Akt inhibitors could completely suppress the Akt pathway in non-vascular tissues which might cause deleterious effects and worsen the disease. Thus, compounds such as MK2206 that provide total Akt inhibition are likely not appropriate for therapy. A pharmacological agent that can fine-tune Akt activity in retinal ECs and other cell types may offer a more suitable PR therapy.

Our pre-clinical and cellular research has demonstrated the therapeutic benefits of TCBN in pulmonary fibrosis [27,55], acute lung injury [28], and COVID-19 [24,56,57] via Akt-dependent and independent mechanisms. These studies also revealed that, unlike MK2206, which completely inhibits Akt activity in cells, TCBN only partially inhibits Akt, which results in a normalizing effect rather than complete inhibition thus avoiding potential side effects. In addition, TCBN is known to increase LDL-receptor expression levels to lower plasma lipid levels [58,59], which could benefit type-2 diabetes patients. Apart from this, TCBN has demonstrated anti-inflammatory [28] and anti-fibrotic [55,60] effects via the generation of regulatory T-cells (Tregs) in mice [28]. Although different adenosine receptors paradoxically respond to inhibitory and activating signals [61], a subset of adenosine receptor activation has been reported to promote wound healing [62]. The ability of TCBN to weakly inhibit Akt along with its other potentially beneficial effects being an adenosine receptor analog makes it a perfect candidate to treat various vascular complications, including PR. The ability of TCBN to inhibit vascular rarefaction and RNV tuft formation in the OIR retinas validates this hypothesis.

The next obvious question is how TCBN mediates its effect in reducing vascular pathology and inflammation in the OIR retina. Whereas similar studies begin treatments before the onset of RNV injury, we initiated TCBN treatment on P14, once after the retinal injury has been induced and the multiple signs of OIR started to appear, and upregulation of many inflammatory genes in the retina [63,64], are evident. This time point was chosen to determine the potential clinical utility of TCBN in the proliferative stage of OIR because most patients with PR seek clinical evaluation at later stages of the disease, only after experiencing vision changes. We observed increased pro-inflammatory cytokine levels such as IL1β, MCP-1, IL6, and TNFα in the OIR retinas, which was attenuated by TCBN treatment. Retinal microglia-generated TNFα and IL1β have been associated with deleterious effects on the retina [65]. MCP-1 is a key chemokine that regulates the migration and infiltration of monocytes/macrophages [66] and elevated MCP-1 levels have been reported in the vitreous humor of patients with PR [67]. The suppression of proinflammatory cytokine expression (IL1β, MCP-1, IL6, and TNFα) and the downregulation of macrophages/microglia, and reduced Müller cell activation in the OIR retina by TCBN are proof of its anti-inflammatory effects.

Increased vascular leakage and RNV are the hallmarks of PR [68]. We and others have shown that Akt1 protects the EC barrier and prevents vascular permeability in various vascular beds via modulation of Cldn5 expression [12,13,19,20,69,70]. Hyperactivation of Akt1 in ECs has also been shown to promote vascular permeability and vascular abnormality in vivo [15], but the status of Cldn5 expression with Akt1 hyperactivation in ECs is unknown. Cldn5 was identified as a major TJ protein of the BRB, which is critical for its integrity [71]. We have recently reported activation of Akt in HRECs by high glucose, advanced glycation end products (AGE), or pro-inflammatory cytokines such as TNFα as well as Akt inhibition by bacterial lipopolysaccharide (LPS) inducing HREC-barrier leakage associated with increased synthesis and pathological intracellular patterning of Cldn5, redistributing it from the cell-cell junctions to the cytosol [18]. Similarly, we have previously reported TNFα-induced Akt hyperactivation and disruption of the cell-cell barrier and reduced Cldn5 expression in dermal EC [72]. In support of these findings, a recent study reports abnormal Cldn5 re-distribution leading to anti-VEGF-resistant diabetic macular edema [73]. An earlier study has also reported differential expression of claudins in retinas during normal development and OIR-induced RNV [74]. Our results combined with these reports indicate that not just Akt1 but fine-tuning of Cldn5 expression and their proper intracellular distribution are also essential for vascular homeostasis. Upregulated αSMA expression in the retinal blood vessels is a likely indication of EC pathology and retinal fibrosis in PR as demonstrated in rodents [48] and human patients [75]. Under pathological conditions, expression of αSMA is elevated in retinal pericytes [76], and Müller cells [77]. Increased expression of Cldn5 and αSMA in the OIR retina, and reversal by TCBN treatment is an indication of the pathological effects of hyperactivation of the Akt1-Cldn5 pathway and demonstrates the efficacy of TCBN to reverse the pathology.

Studies using SD-OCT to analyze retinal layer thickness demonstrated no adverse effect of TCBN on retinal neurons in the OIR retina. Damage to retinal neurons is considered an undesirable side effect of anti-VEGF therapy [78,79]. Others and we have reported neuronal loss and retinal thinning in response to OIR [31,33]. The OIR-induced neuronal death that occurs at the early hyperoxia phase [33] is much higher than that in the hypoxic phase [31], and the peak of neuronal death in the hypoxia phase studied by TUNEL-positive cells is reported to be P14. In contrast, ERG analysis in a different study has shown that neuronal toxicity increases progressively from P12 to P26 in the OIR retina [80]. In either case, since the TCBN treatment in the current study started at P14, this may not be the optimum time point to evaluate whether TCBN can protect neurons in the OIR retina. However, results from our SD-OCT and ERG experiments suggest that TCBN does not cause any adverse effects on retinal neurons. While the neuroprotective effects of TCBN have been reported [81], further studies are needed to determine the impact of TCBN on neuroprotection in retinopathy models.

In summary, our findings demonstrate that TCBN protects against aberrant RNV, restores BRB homeostasis, and reduces retinal inflammation without inducing any adverse effects on the retinal structure and function in a mouse model of OIR. TCBN, which inhibits Akt activity as well as Cldn5 and VEGF expression in HRECs [18], also reduced the pathological expression of αSMA, GFAP, and vimentin in the OIR retina, and prevented vascular leakage without affecting the overall retinal structure and function. Treatment with TCBN prevented the activation of macrophages, microglia, and Müller cells, and reduced the expression of pro-inflammatory cytokines such as IL1β, MCP-1, IL6, and TNFα in the OIR retina. Although the results are promising, our study does have some limitations. This mainly includes a lack of in-depth understanding of how TCBN protects retinal vasculature and inhibits inflammation outside of the modulation of the Akt pathway. Nevertheless, our study reveals the potential benefits of using TCBN in the treatment of PR as a future therapeutic strategy to minimize the damaging effects of PR.

## Supplementary Material

1

## Figures and Tables

**Table 1:** List of antibodies and lectin, and their dilutions used in the study.

Antibody	Catalog Number	Source	Dilution	Experiment
Iba-1, Rabbit	019–19741	Wako	1:200	Immunostaining
Iba1/AIF-1, Rabbit	17198	Cell Signaling	1:1000	Western blot
Glial Fibrillary Acidic Protein, Rabbit	Z033429–2	Agilent Technologies	1:200	Immunostaining
Glial Fibrillary Acidic Protein, Mouse	G6171	Sigma	1:1000	Western blot
α-Smooth Muscle Actin, Mouse	A 2547	Sigma	1:200	Immunostaining
Vimentin, Mouse	sc-6260	Santa Cruz	1:500	Western blot
Isolectin B4 (lectin)	121411	Invitrogen	1:100	Immunostaining
Claudin 5, Mouse	35–2500	Invitrogen	1:100	Immunostaining
F4/80, Rat	ab6640	Abcam	1:100	Immunostaining
TNFα, Mouse	ab1793	Abcam	1:500	Western blot
Phospho-Akt (Ser473), Rabbit	9271	Cell Signaling	1:1000	Western blot
Akt (pan) (C67E7), Rabbit	4691	Cell Signaling	1:500	Western blot
VEGF, Mouse	sc-7269	Santa Cruz	1:200	Western blot
Anti-Rabbit IgG (H+L), Alexa Fluor 488, Donkey	A21206	Invitrogen	1:500	Immunostaining
Anti-Mouse IgG (H+L), Alexa Fluor 555, Donkey	A31570	Invitrogen	1:500	Immunostaining
Anti-Rat IgG (H+L), Alexa Fluor 488	A21208	Invitrogen	1:500	Immunostaining
Albumin, Donkey	16475–1-AP	proteintech	1:1000	Western blot
β-Actin, Mouse	A1978	Sigma	1:1000	Western blot
Anti-Rabbit IgG (H + L)-HRP Conjugate, Goat	1706515	Biorad	1:3000	Western blot
Anti-Mouse IgG (H + L)-HRP Conjugate, Goat	1721011	Biorad	1:3000	Western blot

**Table 2:** PCR Primers were used in the study.

Gene name	Forward primer	Reverse primer
TNFα	GGTCCCCAAAGGGATGAGAA	TGAGGGTCTGGGCCATAGAA
IL1β	CCAAGCAACGACAAAATACC	GTTGAAGACAAACCGTTTTTCC
IL10	GCTCTTACTGACTGGCATGAG	CGCAGCTCTAGGAGCATGTG
MCP-1	GGCTCAGCCAGATGCAGTTAA	CCTACTCATTGGGATCATCTTGCT
IL6	AGACAAAGCCAGAGTCCTTCAG	TGCCGAGTAGATCTCAAAGTGA
IL4	GGTCTCAACCCCCAGCTAGT	GCCGATGATCTCTCTCAAGTGAT
GAPDH	TGGTGAAGGTCGGTGTGAAC	CCATGTAGTTGAGGTCAATGAAGG
HPRT	GAAAGACTTGCTCGAGATGTCATG	CACACAGAGGGCCACAATGT

## Data Availability

Data will be made available on request.

## Abbreviations:

GFAP: glial fibrillary acidic protein
TCBN: triciribine
BRB: blood-retinal barrier
Cldn5: Claudin-5
IL-1β: interleukin-1β
TNF-α: tumor necrosis factor-α
MCP-1: monocyte chemoattractant protein-1
VEGF: vascular endothelial growth factor
HRP: Horseradish peroxidase
IB4: Isolectin B4
OIR: oxygen-induced retinopathy
NFL: nerve fiber layer
INL: inner nuclear layer
OPL: outer plexiform layer
SD-OCT: spectral domain optical coherence tomography
PR: proliferative retinopathy
DR: diabetic retinopathy
HREC: human retinal endothelial cells
RNV: retinal neovascularization
ROP: retinopathy of prematurity
