# Supplementary material for: Triciribine attenuates pathological neovascularization and vascular permeability in a mouse model of proliferative retinopathy
Source: Biomed Pharmacother. Author manuscript; Available in PMC 2023 Jun 1. (PMC10208444; doi:10.1016/j.biopha.2023.114714)
Supplement: 1 [file NIHMS1896479-supplement-1.pdf]

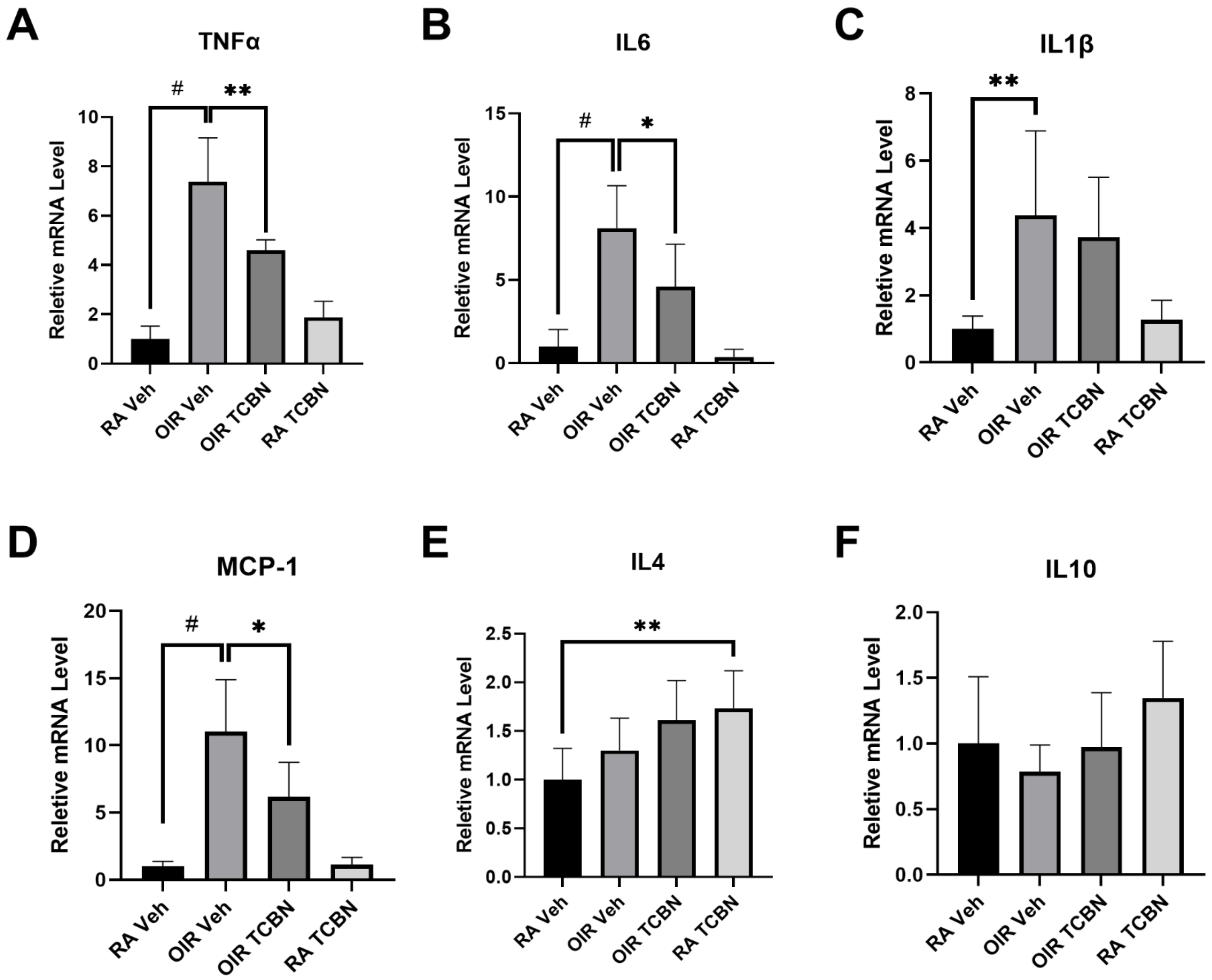

Supplemental Figure S1. Elevated pro-inflammatory cytokines and chemokines in the OIR retinas were suppressed by TCBN. (A-D) Quantitative RT-PCR analysis demonstrating changes in the mRNA levels of pro-inflammatory cytokines and chemokines IL-1β, TNFα, IL-6, and MCP-1, respectively in the retinal samples from RA and OIR mice treated with vehicle or TCBN. (E-F) Quantitative RT-PCR analysis, normalized to hypoxanthine-guanine phosphoribosyl transferase (HPRT), demonstrating changes in the mRNA levels of anti-inflammatory cytokines IL-4 and IL-10, respectively in the above samples. Data are presented as mean ± SD. #$p<0.001$; **$p<0.01$; * $p<0.05$. n = 5-8 per group.

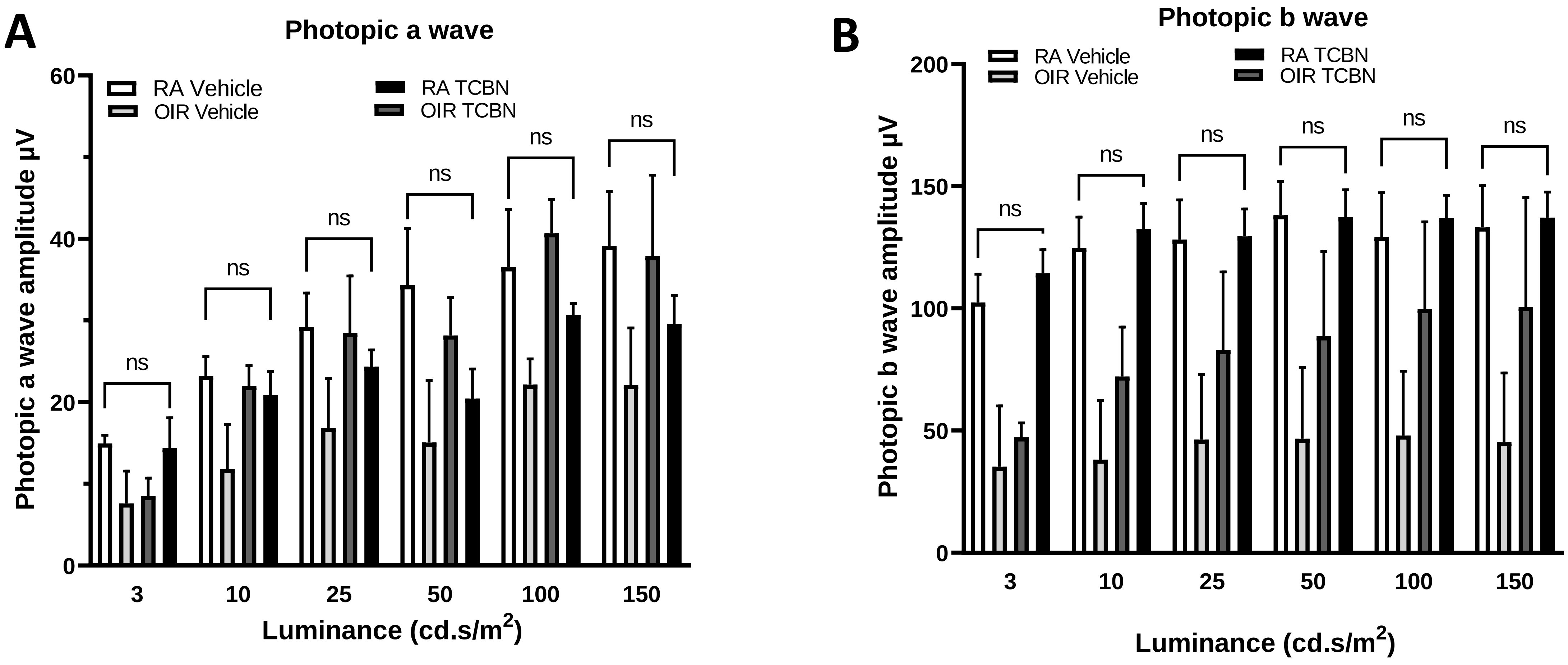

Supplemental Figure S2. TCBN treatment did not affect rod and cone cell function in photopic a and b wave analysis in the mouse retina. Retinal function by light-adapted (photopic) ERG in P26 RA control, RA TCBN, OIR, and OIR+TCBN treated mice were assessed. (A) Photopic a-wave amplitudes and (B) b-wave amplitudes for all four mouse groups plotted at the five light intensities. Changes in photopic a wave and b wave amplitudes were studied at flash intensities ranging from 0.001 to 1.0 candela-seconds per meter squared (cd.s/m2). There were no significant differences between groups for rod or cone responses in P25 RA control and RA TCBN treated mice. Data are shown as mean ± SEM. n= 3-6 per group.
